# 1,2-Di-4-pyridylethane *N*,*N*′-dioxide–acetic acid (1/2)

**DOI:** 10.1107/S1600536809044912

**Published:** 2009-11-04

**Authors:** Elaine P. Boron, Jacqueline M. Knaust

**Affiliations:** a Chemistry Department, 520 North Main St., Meadville, PA 16335, USA

## Abstract

The title compound, C_12_H_12_N_2_O_2_·2C_2_H_4_O_2_, was prepared from 1,2-di-4-pyridylethane, acetic acid, and hydrogen peroxide. The 1,2-di-4-pyridylethane *N*,*N*′-dioxide mol­ecule is located on an inversion center. π–π stacking inter­actions between neighboring 1,2-di-4-pyridylethane *N*,*N*′-dioxide mol­ecules are observed with a centroid–centroid distance of 3.613 Å, an inter­planar distance of 3.317 Å, and a slippage of 1.433 Å. O—H⋯O hydrogen-bonding inter­actions between 1,2-di-4-pyridylethane *N*,*N*′-dioxide and acetic acid mol­ecules result in distinct hydrogen-bonded units made of one *N*-oxide and two acetic acid mol­ecules. These units are then linked into a three-dimensional network through weaker C—H⋯O hydrogen-bonding inter­actions.

## Related literature

For the synthesis of 2,2′-bipyridine *N*,*N*′-dioxide, see: Simpson *et al.* (1963 ▶). For the synthesis of 1,2-di-4-pyridylethane *N*,*N*′-dioxide peroxide disolvate and its use in the synthesis of lanthanide coordination networks, see: Lu *et al.* (2002 ▶). Zhang, Du *et al.* (2004 ▶) and Zhang, Lu *et al.* (2004 ▶) also report the use of 1,2-di-4-pyridylethane *N*,*N*′-dioxide in the preparation of lanthanide coordination networks.
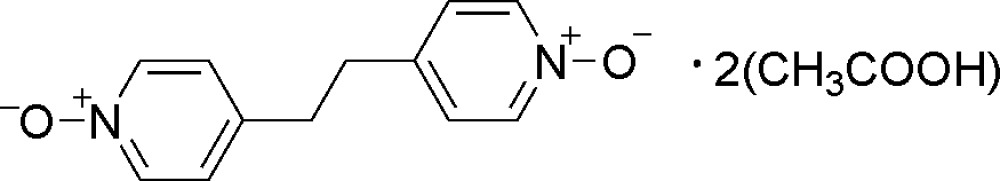


## Experimental

### 

#### Crystal data

C_12_H_12_N_2_O_2_·2C_2_H_4_O_2_
*M* *_r_* = 336.34
Triclinic, 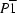

*a* = 7.1109 (6) Å
*b* = 7.1562 (6) Å
*c* = 9.2888 (7) Å
α = 73.719 (1)°
β = 87.508 (1)°
γ = 64.424 (1)°
*V* = 407.62 (6) Å^3^
*Z* = 1
Mo *K*α radiation
μ = 0.11 mm^−1^
*T* = 173 K
0.55 × 0.45 × 0.37 mm

#### Data collection

Bruker SMART APEX CCD diffractometer
Absorption correction: multi-scan (*SADABS*; Bruker, 2001 ▶) *T*_min_ = 0.944, *T*_max_ = 0.962
4857 measured reflections
2446 independent reflections
2228 reflections with *I* > 2σ(*I*)
*R*_int_ = 0.011

#### Refinement

*R*[*F* ^2^ > 2σ(*F* ^2^)] = 0.044
*wR*(*F* ^2^) = 0.133
*S* = 1.09
2446 reflections
114 parameters
H-atom parameters constrained
Δρ_max_ = 0.39 e Å^−3^
Δρ_min_ = −0.25 e Å^−3^

### 

Data collection: *SMART* (Bruker, 2007 ▶); cell refinement: *SAINT-Plus* (Bruker, 2007 ▶); data reduction: *SAINT-Plus*; program(s) used to solve structure: *SHELXS97* (Sheldrick, 2008 ▶); program(s) used to refine structure: *SHELXL97* (Sheldrick, 2008 ▶); molecular graphics: *X-SEED* (Barbour, 2001 ▶); software used to prepare material for publication: *X-SEED*.

## Supplementary Material

Crystal structure: contains datablocks global, I. DOI: 10.1107/S1600536809044912/zl2243sup1.cif

Structure factors: contains datablocks I. DOI: 10.1107/S1600536809044912/zl2243Isup2.hkl

Additional supplementary materials:  crystallographic information; 3D view; checkCIF report

## Figures and Tables

**Table 1:** Hydrogen-bond geometry (Å, °)

*D*—H⋯*A*	*D*—H	H⋯*A*	*D*⋯*A*	*D*—H⋯*A*
O3—H3⋯O1^i^	0.84	1.72	2.5393 (11)	164
C1—H1⋯O2^i^	0.95	2.68	3.3915 (12)	132
C2—H2⋯O3^ii^	0.95	2.45	3.3489 (11)	158
C5—H5⋯O1^iii^	0.95	2.48	3.3341 (12)	149
C6—H6*B*⋯O1^iv^	0.99	2.66	3.6309 (12)	168
C8—H8*C*⋯O1^v^	0.98	2.52	3.3655 (13)	145

Symmetry codes: (i) 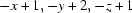; (ii) 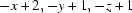; (iii) 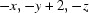; (iv) 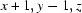; (v) 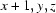.

**Fig. 1.**
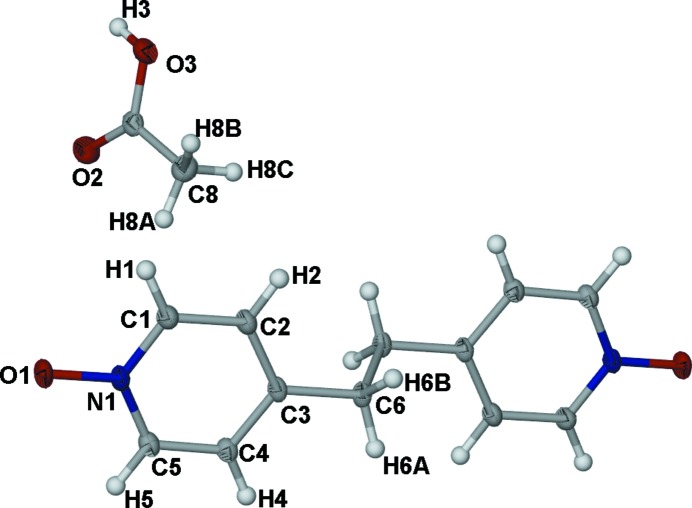
The molecular structure of the title compound with atom labels and 50% probability displacement ellipsoids for non-H atoms.

**Fig. 2.**
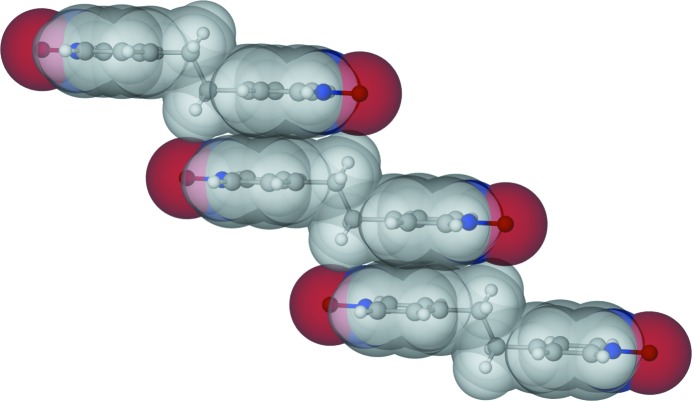
π-π interactions between neighboring 1,2-di-4-pyridylethane N,N'-dioxide molecules.

**Fig. 3.**
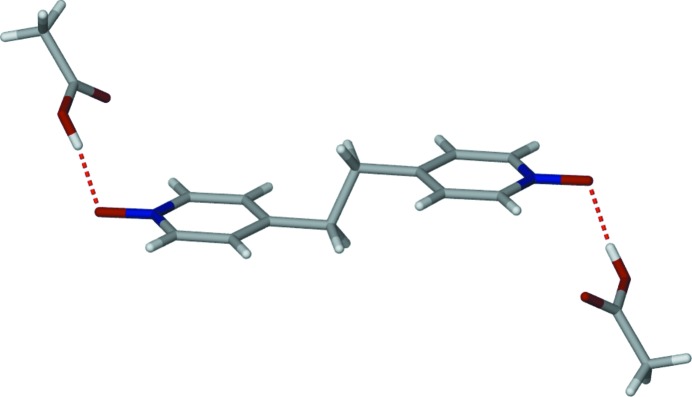
O—H···O hydrogen bonded units made of one 1,2-di-4-pyridylethane N,N'-dioxide molecule and two acetic acid molecules. Hydrogen bonds are shown as dashed lines.

**Fig. 4.**
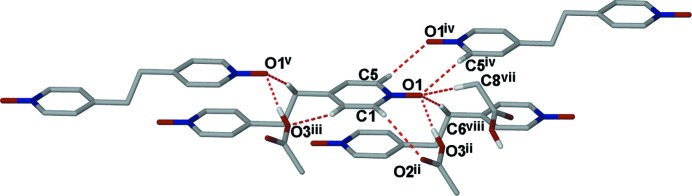
O—H···O and C—H···O hydrogen bonding interactions between 1,2-di-4-pyridylethane N,N'-dioxide and neighboring 1,2-di-4-pyridylethane N,N'-dioxide and acetic acid molecules. Hydrogen bonds are shown as dashed lines. Hydrogen atoms not involved in the hydrogen bonds shown have been omitted for clarity. Symmetry codes: (ii) -x + 1, -y + 2, -z + 1; (iii) -x + 2, -y + 1, -z + 1; (iv) -x, -y + 2, -z; (v) x + 1, y - 1, z; (vii) x - 1, y, z; (viii) x - 1,y + 1, z.

**Fig. 5.**
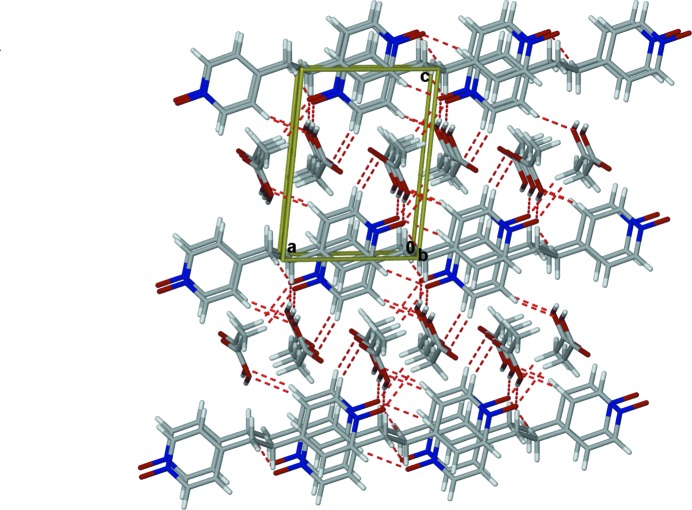
Packing of the title compound viewed down the b axis. Hydrogen bonds are shown as dashed lines.

## supplementary crystallographic information

### Comment

The use of aromatic *N*,*N*'-dioxide ligands in the synthesis of coordination networks has been of recent interest (Lu *et al.* (2002), Zhang, Du *et al.* (2004) and Zhang, Lu *et al.* (2004)). The title compound was prepared using the reaction conditions described by Simpson *et al.* (1963) to prepare 2,2'-bipyridine *N*,*N*'-dioxide. The molar ratios of reactants used to form the title compound were 1:20:3 (1,2-di-4-pyridylethane, acetic acid, and peroxide), and the reaction mixture was heated for 21 h. However, when precipitation of the product did not occur following the addition of acetone as described by Simpson *et al.* (1963), the solution was cooled to 273 K, and crystals of the title compound slowly formed. Lu *et al. *(2002) described the synthesis of 1,2-di-4-pyridylethane *N*,*N*'-dioxide peroxide disolvate using a slightly modified version of the conditions described by Simpson *et al.* (1963). The molar ratios of reactants used by Lu *et al.* (2002) are 1:13:8, and the reaction was heated for 12 h. Lu *et al.* (2002) removed all excess acetic acid and water under vacuum before adding acetone to the resulting oil to precipitate the crude product; the crude product was washed to remove unreacted 1,2-di-4-pyridylethane and recrystallized to give 1,2-di-4-pyridylethane *N*,*N*'-dioxide peroxide disolvate. Presumably, the formation of the acetic acid adduct *versus* the peroxide adduct is due to the difference in reaction and crystallization conditions. The title compound is formed with a high 1,2-di-4-pyridylethane to acetic acid ratio and crystallization directly from the reaction solution. Whereas the peroxide adduct is formed with a high 1,2-di-4-pyridylethane to peroxide ratio and removal of excess acetic acid before crystallization.

The asymmetric unit of the title compound contains half of a 1,2-di-4-pyridylethane *N*,*N*'-dioxide molecule and one acetic acid molecule (Figure 1). The 1,2-di-4-pyridylethane *N*,*N*'-dioxide sits on a center of inversion. π-π stacking interactions with a centroid to centroid distance of 3.6133 Å, an interplanar distance of 3.3171 Å, and a slippage of 1.433 Å. are observed between neighboring N-oxide molecules [symmetry code: -*x* + 1, -*y* + 1, -*z*] (Figure 2). The title compound forms distinct O—H···O hydrogen bonded units made of one N-oxide molecule and two acetic acid molecules (Figure 3). Weaker O—H···O hydrogen bonding interactions are also observed between N-oxide and acetic acid molecules and between neighboring N-oxide molecules (Figure 4). As seen in the packing diagram, the N-oxide and acetic acid molecules are linked into a three-dimensional hydrogen-bonding network (Figure 5).

### Experimental

1,2-Di-4-pyridylethane (11.7918 g, 64.0 mmol), acetic acid (75 ml), and 35% hydrogen peroxide (11.1 ml) were heated at 343–353K (70–80 °C) for 3 h. Additional hydrogen peroxide (7.8 ml) was added, and heating was continued. After an additional 19 h of heating the solution was cooled to room temperature. Crystals formed upon the addition of acetone (1*L*) and cooling to 273 K.

### Refinement

All H atoms were positioned geometrically and refined using a riding model with C—H = 0.95–0.99 Å and with *U*_iso_(H) = 1.2 (1.5 for methyl groups) times *U*_eq_(C), and O—H = 0.84 Å and *U*_iso_(H) = 1.5 times *U*_eq_(O).

### Crystal data

**Table:**

C_12_H_12_N_2_O_2_·2C_2_H_4_O_2_	*Z* = 1
*M**_r_* = 336.34	*F*(000) = 178
Triclinic, *P*1	*D*_x_ = 1.370 Mg m^−^^3^
Hall symbol: -P 1	Mo *K*α radiation, λ = 0.71073 Å
*a* = 7.1109 (6) Å	Cell parameters from 3794 reflections
*b* = 7.1562 (6) Å	θ = 3.2–30.5°
*c* = 9.2888 (7) Å	µ = 0.11 mm^−^^1^
α = 73.719 (1)°	*T* = 173 K
β = 87.508 (1)°	Block, colorless
γ = 64.424 (1)°	0.55 × 0.45 × 0.37 mm
*V* = 407.62 (6) Å^3^	

### Data collection

**Table:**

Bruker SMART APEX CCD diffractometer	2446 independent reflections
Radiation source: fine-focus sealed tube	2228 reflections with *I* > 2σ(*I*)
graphite	*R*_int_ = 0.011
ω scans	θ_max_ = 30.5°, θ_min_ = 2.3°
Absorption correction: multi-scan (*SADABS*; Bruker, 2001)	*h* = −10→10
*T*_min_ = 0.944, *T*_max_ = 0.962	*k* = −10→9
4857 measured reflections	*l* = −13→13

### Refinement

**Table:**

Refinement on *F*^2^	Primary atom site location: structure-invariant direct methods
Least-squares matrix: full	Secondary atom site location: difference Fourier map
*R*[*F*^2^ > 2σ(*F*^2^)] = 0.044	Hydrogen site location: inferred from neighbouring sites
*wR*(*F*^2^) = 0.133	H-atom parameters constrained
*S* = 1.09	*w* = 1/[σ^2^(*F*_o_^2^) + (0.0796*P*)^2^ + 0.0824*P*] where *P* = (*F*_o_^2^ + 2*F*_c_^2^)/3
2446 reflections	(Δ/σ)_max_ = 0.001
114 parameters	Δρ_max_ = 0.39 e Å^−^^3^
0 restraints	Δρ_min_ = −0.25 e Å^−^^3^

### Special details

**Table:**

Geometry. All e.s.d.'s (except the e.s.d. in the dihedral angle between two l.s. planes) are estimated using the full covariance matrix. The cell e.s.d.'s are taken into account individually in the estimation of e.s.d.'s in distances, angles and torsion angles; correlations between e.s.d.'s in cell parameters are only used when they are defined by crystal symmetry. An approximate (isotropic) treatment of cell e.s.d.'s is used for estimating e.s.d.'s involving l.s. planes.
Refinement. Refinement of *F*^2^ against ALL reflections. The weighted *R*-factor *wR* and goodness of fit *S* are based on *F*^2^, conventional *R*-factors *R* are based on *F*, with *F* set to zero for negative *F*^2^. The threshold expression of *F*^2^ > σ(*F*^2^) is used only for calculating *R*-factors(gt) *etc*. and is not relevant to the choice of reflections for refinement. *R*-factors based on *F*^2^ are statistically about twice as large as those based on *F*, and *R*- factors based on ALL data will be even larger.

### Fractional atomic coordinates and isotropic or equivalent isotropic displacement parameters (Å^2^)

**Table:**

	*x*	*y*	*z*	*U*_iso_*/*U*_eq_	
O1	0.13750 (10)	0.89616 (12)	0.18453 (8)	0.02574 (15)	
O2	0.65037 (13)	1.15878 (12)	0.46617 (9)	0.03371 (18)	
O3	0.86230 (12)	0.87364 (12)	0.65134 (8)	0.02891 (16)	
H3	0.8470	0.9693	0.6925	0.043*	
N1	0.32388 (11)	0.79711 (12)	0.13545 (9)	0.019	
C1	0.48607 (14)	0.64120 (15)	0.23478 (10)	0.022	
H1	0.4674	0.6045	0.3391	0.026*	
C2	0.67866 (13)	0.53522 (15)	0.18518 (10)	0.02148 (17)	
H2	0.7919	0.4267	0.2558	0.026*	
C3	0.70835 (13)	0.58611 (14)	0.03222 (10)	0.01794 (15)	
C4	0.53728 (13)	0.74729 (14)	−0.06672 (10)	0.01899 (16)	
H4	0.5520	0.7860	−0.1717	0.023*	
C5	0.34614 (13)	0.85159 (14)	−0.01355 (10)	0.01970 (16)	
H5	0.2308	0.9614	−0.0819	0.024*	
C6	0.91767 (13)	0.46745 (14)	−0.02202 (10)	0.01940 (16)	
H6A	0.9022	0.5004	−0.1329	0.023*	
H6B	0.9662	0.3095	0.0222	0.023*	
C7	0.76154 (14)	0.96654 (16)	0.51521 (10)	0.02359 (18)	
C8	0.80145 (17)	0.80587 (18)	0.42910 (12)	0.0301 (2)	
H8A	0.6925	0.8686	0.3456	0.045*	
H8B	0.7993	0.6741	0.4963	0.045*	
H8C	0.9386	0.7700	0.3892	0.045*	

### Atomic displacement parameters (Å^2^)

**Table:**

	*U*^11^	*U*^22^	*U*^33^	*U*^12^	*U*^13^	*U*^23^
O1	0.0129 (3)	0.0322 (4)	0.0298 (3)	−0.0051 (3)	0.0056 (2)	−0.0140 (3)
O2	0.0348 (4)	0.0244 (4)	0.0278 (4)	−0.0039 (3)	0.0014 (3)	−0.0014 (3)
O3	0.0253 (3)	0.0244 (3)	0.0276 (4)	−0.0027 (3)	−0.0033 (3)	−0.0063 (3)
N1	0.012	0.020	0.024	−0.005	0.003	−0.008
C1	0.017	0.024	0.021	−0.006	0.001	−0.006
C2	0.0149 (4)	0.0216 (4)	0.0235 (4)	−0.0041 (3)	0.0004 (3)	−0.0061 (3)
C3	0.0131 (3)	0.0171 (4)	0.0241 (4)	−0.0063 (3)	0.0025 (3)	−0.0073 (3)
C4	0.0157 (4)	0.0184 (4)	0.0214 (4)	−0.0069 (3)	0.0024 (3)	−0.0046 (3)
C5	0.0148 (3)	0.0184 (4)	0.0233 (4)	−0.0056 (3)	0.0010 (3)	−0.0047 (3)
C6	0.0136 (3)	0.0195 (4)	0.0260 (4)	−0.0064 (3)	0.0038 (3)	−0.0096 (3)
C7	0.0177 (4)	0.0260 (4)	0.0224 (4)	−0.0078 (3)	0.0058 (3)	−0.0036 (3)
C8	0.0292 (5)	0.0314 (5)	0.0272 (5)	−0.0109 (4)	0.0061 (4)	−0.0093 (4)

### Geometric parameters (Å, °)

**Table:**

O1—N1	1.3358 (9)	C3—C6	1.5079 (11)
O2—C7	1.2107 (12)	C4—C5	1.3859 (11)
O3—C7	1.3231 (12)	C4—H4	0.9500
O3—H3	0.8400	C5—H5	0.9500
N1—C5	1.3506 (12)	C6—C6^i^	1.5410 (17)
N1—C1	1.3530 (12)	C6—H6A	0.9900
C1—C2	1.3811 (12)	C6—H6B	0.9900
C1—H1	0.9500	C7—C8	1.5021 (14)
C2—C3	1.3950 (13)	C8—H8A	0.9800
C2—H2	0.9500	C8—H8B	0.9800
C3—C4	1.3951 (12)	C8—H8C	0.9800
			
O1—N1—C1	119.76 (8)	C1—C2—H2	119.7
C5—N1—C1	120.99 (8)	C2—C1—H1	119.8
O1—N1—C5	119.24 (7)	C3—C2—H2	119.7
N1—C1—C2	120.31 (8)	C3—C4—H4	119.6
N1—C5—C4	120.00 (8)	C3—C6—H6A	109.3
C1—C2—C3	120.58 (8)	C3—C6—H6B	109.3
C2—C3—C4	117.43 (8)	C4—C5—H5	120.0
C3—C4—C5	120.70 (8)	C5—C4—H4	119.6
C4—C3—C6	122.07 (8)	C6^i^—C6—H6A	109.3
C2—C3—C6	120.50 (8)	C6^i^—C6—H6B	109.3
C3—C6—C6^i^	111.43 (8)	C7—C8—H8A	109.5
O2—C7—O3	123.73 (10)	C7—C8—H8B	109.5
O2—C7—C8	124.14 (9)	C7—C8—H8C	109.5
O3—C7—C8	112.13 (8)	H6A—C6—H6B	108.0
N1—C1—H1	119.8	H8A—C8—H8B	109.5
N1—C5—H5	120.0	H8A—C8—H8C	109.5
C7—O3—H3	109.5	H8B—C8—H8C	109.5

Symmetry codes: (i) −*x*+2, −*y*+1, −*z*.

### Hydrogen-bond geometry (Å, °)

**Table:**

*D*—H···*A*	*D*—H	H···*A*	*D*···*A*	*D*—H···*A*
O3—H3···O1^ii^	0.84	1.72	2.5393 (11)	164
C1—H1···O2^ii^	0.95	2.68	3.3915 (12)	132
C2—H2···O3^iii^	0.95	2.45	3.3489 (11)	158
C5—H5···O1^iv^	0.95	2.48	3.3341 (12)	149
C6—H6B···O1^v^	0.99	2.66	3.6309 (12)	168
C8—H8C···O1^vi^	0.98	2.52	3.3655 (13)	145

Symmetry codes: (ii) −*x*+1, −*y*+2, −*z*+1; (iii) −*x*+2, −*y*+1, −*z*+1; (iv) −*x*, −*y*+2, −*z*; (v) *x*+1, *y*−1, *z*; (vi) *x*+1, *y*, *z*.
